# Internet Usage by Parents Prior to Seeking Care at a Pediatric Emergency Department: Observational Study

**DOI:** 10.2196/ijmr.5075

**Published:** 2017-09-28

**Authors:** Purvi L Shroff, Rebecca W Hayes, Pradeep Padmanabhan, Michelle D Stevenson

**Affiliations:** ^1^ Pediatric Emergency Medicine Associates Atlanta, GA United States; ^2^ Family Residency Program St. Louis University Belleville, IL United States; ^3^ Division of Pediatric Emergency Medicine Department of Pediatrics University of Louisville Louisville, KY United States

**Keywords:** Internet, emergency department, decision making

## Abstract

**Background:**

Little is known about how parents utilize medical information on the Internet prior to an emergency department (ED) visit.

**Objective:**

The objective of the study was to determine the proportion of parents who accessed the Internet for medical information related to their child’s illness in the 24 hours prior to an ED visit (IPED), to identify the websites used, and to understand how the content contributed to the decision to visit the ED.

**Methods:**

A 40-question interview was conducted with parents presenting to an ED within a freestanding children’s hospital. If parents reported IPED, the number and names of websites were documented. Parents indicated the helpfulness of Web-based content using a 100-mm visual analog scale and the degree to which it contributed to the decision to visit the ED using 5-point Likert-type responses.

**Results:**

About 11.8 % (31/262) reported IPED (95% CI 7.3-5.3). Parents who reported IPED were more likely to have at least some college education (*P*=.04), higher annual household income (*P=*.001), and older children (*P=*.04) than those who did not report IPED. About 35% (11/31) could not name any websites used. Mean level of helpfulness of Web-based content was 62 mm (standard deviation, SD=25 mm). After Internet use, some parents (29%, 9/31) were more certain they needed to visit the ED, whereas 19% (6/31) were less certain. A majority (87%, 195/224) of parents who used the Internet stated that they would be somewhat likely or very likely to visit a website recommended by a physician.

**Conclusions:**

Nearly 1 out of 8 parents presenting to an urban pediatric ED reported using the Internet in the 24 hours prior to the ED visit. Among privately insured, at least one in 5 parents reported using the Internet prior to visiting the ED. Web-based medical information often influences decision making regarding ED utilization. Pediatric providers should provide parents with recommendations for high-quality sources of health information available on the Internet.

## Introduction

Many parents seek advice from at least one source prior to presenting to a pediatric emergency department (ED) [1-5]. Parents commonly utilize the Internet to learn about pediatric health problems [1,2,5-16]. However, many websites do not present accurate evidence-based medical information or advice [6,9,17-20]. Deciphering health information and verifying accuracy can be a difficult task for parents [5,11,12,21-24]. Therefore, pediatric health care providers need to understand how families access and utilize medical information obtained on the Internet.

To our knowledge, only two studies have examined Internet use specific to parents who present to the pediatric ED; however, neither study was conducted in the United States [2,8]. Little is known about the websites that parents choose to visit or how this information contributes to the decision to visit the pediatric ED. The objective of this study was to determine the percentage of parents who search the Internet for medical information related to their child’s presenting complaint in the 24 hours prior to the ED visit. Furthermore, we aimed to identify the websites accessed by parents and to study the degree to which the information found on the Internet contributes to the decision to visit the ED. We hypothesized that 20% of study participants would have searched the Internet in the 24 hours prior to their ED visit.

## Methods

### Conducting Structured Interviews

We conducted structured interviews from a convenience sample of parents in the ED from July 2010 to July 2011. This facility is a freestanding, urban, tertiary care children’s hospital with approximately 54,000 annual visits. English-speaking parents or legal guardians of children presenting for care in either the main ED or the fast track area from 6:00 AM to 12:00 AM were eligible. Parents of children triaged at an emergency severity index (ESI) level of 1 [25], evaluated for abuse or neglect, transported by ambulance from the scene of a significant trauma, or transferred from a referring facility were excluded. Parents who had not been significantly involved in the decision to bring their child to the ED (eg, a child who presented directly from school), had registered more than one child to be seen, or had previously participated in the study were also excluded. If more than one parent was present, we asked for a volunteer and administered the interview only to that parent.

Eligible parents were interviewed in the exam rooms by either the primary investigator or a single research assistant trained by the primary investigator, within a window of time during which patient care would not be hindered. We utilized a 40-item paper questionnaire, which was developed by the research team and finalized after a short pilot study of 20 parents. After obtaining informed consent, the interviewer read the questions from the questionnaire and documented the parent’s answers on the paper form. A combination of closed- and open-ended questions covered demographics, details of the ED visit, resources utilized (Internet and non-Internet), and accessibility to Internet. For all parents who identified themselves as Internet users, we were interested in their health information–seeking behavior. To understand this, we asked about the frequency of Internet use for any topic concerning their child’s health, their awareness of certain pediatric health–focused websites, and their interest in visiting websites recommended to them by a physician. Parents who reported accessing the Internet for medical information related to their child’s presenting complaint in the 24 hours preceding the ED visit (Internet use prior to the ED visit, or IPED) were asked the number and names of websites used, and the helpfulness of the content using a visual analog scale (VAS) [26], and how the information contributed to the decision to visit the ED using 5-point Likert-type response questions. For the question utilizing the VAS, the parents marked their answers on the paper questionnaire. One paper questionnaire was filled out per parent interviewed. The average amount of time for completion of the questionnaire was 10 min. We also conducted the same structured interview among a smaller sample of parents presenting to our suburban satellite facility, which opened after enrollment had begun at our main urban site. As the patient population at this location is different from the urban site, we enrolled a small sample of patients to determine whether a larger study at this separate facility with a different patient population would be worthwhile. Taking this into consideration, pertinent results and analysis from this sample are presented. This suburban facility is a 24-hour full-service pediatric ED, staffed by the same faculty as our main study site, with an approximate annual volume of 15,000 patients. The data showing the differences at the two sites are available to the reader upon request. We enrolled from August 2011 to September 2011, employing the same inclusion and exclusion criteria. The percentage of parents who reported IPED was calculated separately for this site.

### Sample Size Derivation and Statistical Analysis

We estimated that there would be at least 20,000 potential participants during the study period. Using a CI of 95% with a margin of error of ±5%, we calculated a sample size ranging from 243 to 377 (using the hypothesis that 20% of parents would have IPED or a worst-case scenario of 50%). General descriptive statistics such as frequency, proportions, student *t* test (continuous variables), chi-square (categorical variables) test, and the Mann-Whitney *U* test were employed to analyze survey data using the Statistical Package for the Social Sciences (SPSS) Statistics version 19 (IBM Corp). A *P* value of <.05 was considered statistically significant. This study was reviewed and approved by our local institutional review board.

## Results

### Main Urban Site

#### Demographic Data

At the main urban hospital, we approached 326 parents, of whom 47 were determined to be ineligible (referred from another facility or not significantly involved in the decision to bring the child to ED); 17 declined to participate. Of the remaining 262 parents interviewed, 81% were mothers, and 96% of the study population reported that their child had a primary care physician. Descriptive data regarding all study participants at the urban site are presented in Table 1. The demographic distribution of our study population was consistent with the overall distribution of patients seen at this facility during the calendar year 2011 (data available upon request).

#### Non-Internet Resources Utilized

Over half of parents (56.1%, 147/262) had consulted at least one non-Internet resource prior to deciding to bring their child to the ED. The most frequently consulted resource was the patient’s primary care doctor (45.6%, 67/147), followed by a family member (34.7%, 51/147).

#### Internet Accessibility

Of all participants at the urban site, 85.5% (224/262) identified themselves as Internet users, of whom 94.2% (211/224) reported having daily access to the Internet. The most frequently used device was a computer located in the parents’ home. A majority (52.7%, 118/224) of Internet users reported that they used 2 or more devices for Internet access, and 55.8% (125/224) reported using a mobile phone.

#### Primary Objective: Internet Use Prior to ED Visit

Among all 262 parents interviewed, the percentage that reported IPED was 11.8% (31/262; 95% CI 8.5-16.3). Among only the parents with access to the Internet (n=224), 13.8% (31/224; 95% CI 9.9-18.9) reported IPED. Table 2 details the characteristics of all Internet users, grouped according to the presence or lack of IPED. Parents who reported IPED were significantly more likely to have at least some college education, had higher annual household income, had older children, and were slightly older themselves than those who did not report IPED. The most common chief complaints among those who reported IPED were abdominal pain, vomiting, cough, and fever.

Although insurance status was not significantly associated with IPED among only parents with access to the Internet (n=224), it was found that among all participants who had insurance status available (n=261), parents who had a child covered by private insurance showed a higher propensity (12/62, 19%) toward IPED compared with those covered by nonprivate insurance (19/199, 9.5%). This comparison was statistically significant using chi-square (*P*=.04).

#### Websites Used and Influence on Decision Making

We derived this analysis from the 31 parents who reported IPED. Within this group, 48% (15/31) reported using only one website. When asked to name the websites that they consulted, 35% (11/31) of parents were unable to name any of the websites used. Another 26% (8/31) were able to name only some of the websites used. When parents were able to recall the names, the most commonly named websites were WebMD (10/31) and Wikipedia (6/31).

Parents gave a mean score of 62 mm (SD 25 mm) on the VAS, favoring helpfulness of the Internet content. When asked to what degree the content contributed to their decision to visit the ED, 29% (9/31) of parents said that they were *more certain* they needed to bring their child to the ED after viewing the Internet content, and 19% (6/31) said they were *less certain*. For 16% (5/31), the Internet was the only resource that they consulted. The remainder 84% (26/31) had also consulted at least one non-Internet resource. Many of these parents (65%, 20/31) stated that they were already either somewhat certain or very certain that they needed to bring their child to the ED prior to consulting any resources.

#### IPED With Past ED Visit

Among all Internet users (n=224), 20 parents reported IPED in conjunction with an ED visit in the preceding 3 months. Within this group, 4 parents had decided to bring their child to the ED directly based on medical information found on the Internet, and 9 parents stated they had avoided an ED visit directly based on medical information found on the Internet.

#### General Health Information–Seeking Behavior

About 52.2% (117/224) of all Internet users reported at least one episode of Internet use for general pediatric health information in the preceding 3 months. Almost 14.7% (33/224) reported using the Internet more than 6 times for this purpose. Of these, the majority (135/224, 60.2%) were unable to recall specific names of websites used, but reported that they used a search engine such as Google to find information. When specific websites were named, they ranged from product websites (such as Gerber, Fisher Price, and Huggies), to medical establishment websites (such as Cleveland Clinic, Mayo Clinic, and Johns Hopkins), parenting lifestyle websites (such as BabyCenter), or national association websites (National Down Syndrome Society and Epilepsy Foundation), in addition to the commonly named WebMD and Wikipedia.

We were interested to know how often in the preceding 3 months Internet users utilized a short list of websites that we as a research team considered good sources for pediatric health information—healthychildren.org (sponsored by the American Academy of Pediatrics), CDC.gov (Centers for Disease Control and Prevention), kidshealth.org, and our local children’s hospital website. Overall, 10.2% (23/224) reported visiting healthychildren.org, 15.6% (35/224) visited CDC.gov, 9.4% (21/224) visited kidshealth.org, and 15.6% (35/224) visited our local children’s hospital website. Finally, when all Internet users were asked about their interest in utilizing websites recommended to them by a physician, the majority of users (195/224, 87.1%) stated that they would be somewhat likely or very likely to visit the physician-recommended website.

**Table 1:** Demographic data: all participants from urban site (N=262).

Demographic characteristic		n (%) or median (interquartile range)	
**Relationship to child, n (%)**			
	Mother or female guardian		220 (84)
	Father or male guardian		42 (16)
**Age of parent in years, median (interquartile range)**		31 (25-37)	
**Age of child in years, median (interquartile range)**		4 (1.3-11)	
**Race of parent, n (%)**			
	White		135 (52)
	Nonwhite		126 (48)
**Race of child, n (%)^a^**			
	White		124 (47)
	Nonwhite		137 (52)
**Education of parent, n (%)**			
	Some high school		34 (13)
	Completed high school or General Educational Development (GED)		85 (32)
	Some college		97 (37)
	Completed college		32 (12)
	Advanced degree or beyond		14 (5)
**Gross household income in US dollars, n (%)**			
	Less than $25,000		125 (48)
	$25,000-$50,000		74 (28)
	$50,000-$75,000		21 (8)
	$75,000-$100,000		11 (4)
	Greater than $100,000		12 (5)
**Insurance of child, n (%)^a^**			
	Private		62 (24)
	Nonprivate^b^		199 (76)
**Triage classification, n (%)**			
	ESI^c^ 2		54 (21)
	ESI 3		145 (55)
	ESI 4		63 (24)
**Day of enrollment, n (%)**			
	Weekend		17 (7)
	Weekday		245 (94)
**Time of enrollment, n (%)**			
	6 AM - 12 PM		15 (6)
	12 PM - 6 PM		159 (61)
	6 PM - 12 AM		88 (34)
**Disposition, n (%)**			
	Discharge		207 (79)
	Admit		55 (21)

^a^Some data were missing for a few participants (eg, child race and insurance).

^b^Nonprivate includes government insurance, self-pay, and no insurance.

^c^ESI: emergency severity index.

**Table 2:** Internet users (N=224).

Demographic characteristic		Used Internet in 24 hours prior to ED^a^ (n=31), n (%)		Did not use Internet prior to ED (n=193), n (%)		*P* value	
**Relationship to child, n (%)**							
	Mother or female guardian		25 (13)		166 (87)		.43
	Father or male guardian		6 (18)		27 (82)		
**Median age of parent in years**		35		31		.008	
**Median age of child in years**		9		3		.04	
**Race of parent, n (%)**							
	White		20 (17)		95 (83)		.12
	Nonwhite		11 (10)		97 (90)		
**Race of child, n (%)**							
	White		18(17)		88 (83)		.21
	Nonwhite		13 (11)		104 (89)		
**Education of parent, n (%)**							
	High school grad or less		7 (8)		81 (92)		.04
	Some college or more		24 (18)		112 (82)		
**Gross household income in US dollars, n (%)**							
	Less than $25,000		11 (11)		92 (89)		.001
	$25,000-$100,000		12 (13)		84 (87)		
	Greater than $100,000		6 (55)		5 (46)		
**Insurance of child, n (%)**							
	Private		12 (20)		47 (80)		.096
	Nonprivate^b^		19 (12)		145 (88)		
**Triage classification, n (%)**							
	ESI^c^ 2		4 (9)		43 (91)		.46
	ESI 3		19 (16)		100 (84)		
	ESI 4		8 (14)		50 (86)		
**Day of enrollment, n (%)**							
	Weekend		3 (20)		12 (80)		.47
	Weekday		28 (13)		181 (87)		
**Time of enrollment, n (%)**							
	6 AM - 12 PM		3 (21)		11 (79)		.56
	12 PM - 6 PM		20 (15)		118 (85)		
	6 PM - 12 AM		8 (11)		64 (89)		
**Disposition, n (%)**							
	Discharge		23 (13)		152 (87)		.57
	Admit		8 (16)		41 (84)		

^a^ED: emergency department.

^b^Nonprivate includes government insurance, self-pay, and no insurance.

^c^ESI: emergency severity index.

**Table 3:** Suburban site Internet users (N=49).

Demographic characteristic		Used Internet in 24 hours prior to ED^a^ (n=14), n (%)		Did not use Internet prior to ED (n=35), n (%)		*P* value	
**Relationship to child, n (%)**							
	Mother or female guardian		12 (29)		30 (71)		>.99
	Father or male guardian		2 (29)		5 (71)		
**Median age of parent in years**		36		33		.68	
**Median age of child in years**		4.5		4		.82	
**Race of parent, n (%)**							
	White		10 (24)		31 (76)		.20
	Nonwhite		4 (50)		4 (50)		
**Race of child, n (%)**							
	White		10 (27)		27 (73)		.72
	Nonwhite		4 (33)		8 (67)		
**Education of parent, n (%)**							
	High school grad or less		2 (17)		10 (83)		.46
	Some college or more		12 (32)		25 (68)		
**Gross household income in US dollars, n (%)**							
	Less than $25,000		0 (0)		7 (100)		.18
	$25,000-$100,000		8 (33)		16 (67)		
	Greater than $100,000		5 (36)		9 (64)		
**Insurance of child, n (%)**							
	Private		13 (37)		22 (63)		.04
	Nonprivate^b^		1 (7)		13 (93)		
**Triage classification, n (%)**							
	ESI^c^ 2		0 (0)		1 (100)		.08
	ESI 3		10 (48)		11 (52)		
	ESI 4		3 (13)		20 (87)		
	ESI 5		1 (25)		3 (75)		
**Day of enrollment, n (%)**							
	Weekend		4 (22)		14 (78)		.53
	Weekday		10 (32)		21 (68)		
**Time of enrollment, n (%)**							
	6 AM - 12 PM		0 (0)		1 (100)		.73
	12 PM - 6 PM		1 (20)		4 (80)		
	6 PM - 12 AM		13 (30)		30 (70)		

^a^ED: emergency department.

^b^Nonprivate includes government insurance, self-pay, and no insurance.

^c^ESI: emergency severity index.

### Suburban Site Results

Separately, we interviewed 49 parents from our suburban ED. With regard to the overall demographics, parents here reported higher income levels and education levels, and a larger percentage (35/49, 71%) of children was privately insured. All parents here identified themselves as Internet users. The percentage that reported IPED was 29% (14/49; 95% CI 17.9-42.4). A comparison of demographic characteristics between parents who reported and who did not report IPED revealed that children of parents reporting IPED were more likely to have private insurance (*P*=.04, Table 3). In total, 43% (6/14) used only one website, and 29% (4/14) were unable to name any of the websites consulted. WebMD and Wikipedia were the most commonly named websites. When asked the degree to which the content contributed to their decision to visit the ED, 36% (5/14) of parents said that they were more certain they needed to bring their child to the ED, and 14% (2/14) said they were less certain. Almost all (48/49) Internet users reported that they would be very likely or somewhat likely to visit a website recommended to them by a physician.

## Discussion

### Principal Findings

We found that 1 in 8, or about 11.8% (31/262), of all parents who presented to an urban tertiary care ED had used the Internet for medical information in the 24 hours prior to the visit. To the best of our knowledge, this is the first such study performed in the United States. In 2006, Goldman and Macpherson [8] reported that 8.5% of parents presenting to a tertiary care pediatric ED in Toronto, Ontario, Canada, had searched the Internet for health-related information immediately prior to the ED visit. In 2008, Khoo et al [2] reported that 6% of parents presenting to a tertiary care pediatric ED in Melbourne, Australia, had searched the Internet immediately prior to the ED visit. From 2000 to 2010, Internet access in US homes doubled from 35% to 71% [27]. Moreover, almost half of all US mobile phone subscribers by February 2012 owned smartphones [28]. Data from both the Goldman and Khoo studies were collected in 2003 and 2007, respectively, and likely did not account for the rapid expansion of Internet access points (smartphones and electronic readers) since that time. As such, we expected the percentage of IPED from our study to be higher than that reported by Goldman and Khoo.

On the basis of statistically significant differences in education, income, and insurance status, our study findings suggest that parents from higher socioeconomic status (SES) are more likely to search the Internet prior to coming to the ED. Although our main study (not including the suburban site) yielded an IPED percentage that is higher than the previously reported estimates in the Goldman and Khoo studies, it is below our hypothesis of 20%. Interestingly, if the data are analyzed with respect to insurance status as a measure of SES, that is private insurance representing higher SES and nonprivate representing lower SES, 19% (12/62) of privately insured reported IPED, which very closely estimates our hypothesis. This finding at our main study site is further supported by the results from our suburban site, which sees a larger percentage of higher SES (eg, 71% privately insured), and yielded an IPED of 29% in a small sample.

In contrast, IPED among patients without private insurance at our main site was only 9.5% (19/199). Further research would be needed to clarify the factors that contribute to this difference in the lower SES group. According to our data, this finding is not entirely because of the lack of access to Internet; in fact, about 82.4% (103/125) of parents with an income level of less than $25,000 reported access to the Internet, 95% of whom had daily access to the Internet, with many accessing the Internet on computers in their own homes. Knapp et al [22] reported similar rates of Internet access among low-income parents in Florida. Across the United States, increases in broadband access from 2008 to 2009 were largely fueled by a 34% increase in access among US households with an annual income of less than $30,000 [29]. Parents in a comparable income bracket (annual income of less than $25,000) accounted for 47.7% (125/262) of our study participants at the main site. Within the nonprivate insurance group, 49% (98/199) had completed a high school education and had at least some college education. Prior studies have reported higher Internet use for general health information among more educated parents [16,22]. Further research is warranted to examine the role of education in IPED.

Our study also adds to the literature by reporting which websites parents access in their efforts to decide whether an ED visit is warranted. We found that when parents were able to recall the names of websites, WebMD and Wikipedia were the two most commonly named. Parents overwhelmingly reported using a search engine such as Google to generate a list of potential resources. It is not surprising that WebMD and Wikipedia were the most commonly recalled names, both with IPED, as well as for use with general health information. When search terms such as “vomiting,” “cough,” “belly pain in children,” and “meningitis” are entered into Google, WebMD and Wikipedia consistently appear in the top 5, if not the top 2, search results (search performed by author on November 21, 2016). This suggests that pediatric health–focused sites should aim at positioning their website at the top of a generated list of search results.

Although WebMD and Wikipedia were frequently mentioned, the more interesting and telling piece of information that we uncovered with regard to website identification is that a large number of parents (19/31, 61%) were unable to recall the specific names of websites used. This holds true not only in the group that reported IPED but also among parents who reported Internet utilization for general health concerns (60.2% could not name the websites used). This finding is important because it suggests that parents are not discerning information seekers when it comes to health information on the Internet, or do not know how to be. This is in contrast to the non-Internet resources utilized, where parents instinctively trusted their primary care provider or a family member for advice. There is little published data on how parents choose which websites to consult prior to seeking care for their child, although one recent Austrian study found that parents are most likely to visit a website run by doctors when seeking care in an outpatient clinic for an acute illness [15].

Similar to a recent Canadian study, our data suggest that parents often do not know where to look for good health information [21]. This is implied by the relatively low percentage of parents who visited websites that our study team believed had reliable pediatric health information, such as healthychildren.org or cdc.gov. Similar to other studies, our study also suggests that parents may be unaware of what constitutes good health information (eg, evidence-based information vs anecdotal reports) or how to identify reputable websites [21,22]. Further research is warranted to gain a better understanding of how parents decide what sources of information to trust on the Internet.

In keeping with prior studies, our study results also suggest that parents have some difficulty interpreting or putting into context information found on the Internet [11,12,23,24]. On average, parents found the information helpful to the understanding of their child’s condition, but responses ranged from 0 to 100 on the VAS. In fact, several parents admitted that they were less certain that they needed to bring their child to the ED after consulting the Internet, yet they still did so. For ED providers, this indicates that they may encounter patients brought to the ED unnecessarily based on Internet guidance and, conversely, that patients who may need to come for evaluation may not actually come in. If parents have consulted the Internet, they may have certain preconceived expectations about the ED visit, and if this differs from the actuality, it could affect their perception of the quality of medical care received from the provider or the facility and their overall satisfaction with the ED visit. Thus, it is important for ED providers to recognize that many parents are using the Internet for guidance regarding ED visits.

Another important finding was the overwhelming interest from parents (87.1%) in receiving guidance from a physician about appropriate medical websites. Physicians should recognize this need among our patients’ families and be prepared to offer guidance on websites that provide accurate evidence-based and helpful information. Professional medical organizations should assist providers through approval of their own sources of medical advice or endorsement of others.

Salo et al [30] reported high interest among adult ED patients in receiving website recommendations by physicians, along with discharge paperwork. Further study of the utility of interventions such as providing a list of condition-specific medical websites along with written discharge instructions is warranted in the pediatric ED population.

### Limitations

Our study has several limitations. We enrolled a convenience sample of parents, and we relied on their ability to recall information over 3 months. Although our survey questions were not validated against actual Internet use by parents, the structured interview format allowed for collection of content data beyond search engine names. Only English-speaking parents were enrolled. Of note, at our facility, non-English-speaking parents comprise less than 5% of parents seen in the ED, and translator services are only available by phone. Due to the inherent study design, we were unable to sample parents who chose not to visit the ED. To address this limitation, we asked participants about past instances when they decided not to bring their child to the ED based directly on information they found on the Internet. As a research team, we chose certain websites for inclusion in the survey based upon our own experience and did not use any type of certification criteria such as the HONcode [31]. An in-depth quality review of all websites visited by parents was beyond the scope of this study. Finally, our study was performed at a single center where the majority of children are publicly insured. Thus, our findings may have limited application in other settings. Although we enrolled a sample of 49 parents at our satellite facility, it is a relatively small sample, and a larger study could provide more insight about this patient population. It would be beneficial to replicate our findings in other settings as handheld devices and broadband availability continues to rise.

### Conclusions

Nearly one out of 8 parents used the Internet as a resource for medical information in the 24 hours prior to an ED visit. Among privately insured children, at least one in 5 parents used the Internet prior to the ED visit. For these parents, Web-based medical information appears to influence understanding and decision making with regard to ED utilization. Pediatric health care providers should facilitate access to high-quality electronic health care information because parents are interested in Web-based sources for pediatric health information recommended by physicians.

## Abbreviations

CDC: Centers for Disease Control and Prevention
ED: emergency department
ESI: emergency severity index
GED: General Educational Development
IPED: Internet use prior to an ED visit
SD: standard deviation
SES: socioeconomic status
SPSS: Statistical Package for the Social Sciences
VAS: visual analog scale
